# Propaganda: The Effort Towards Union

**Published:** 1918-04-06

**Authors:** 


### NURSING AFFAIRS IN IRELAND.

### Propaganda: The Effort Towards Union.

Every practical nurse should ask herself two questions: first, what it is that she most desires in connection with her employment? and, secondly, by what possibility that can be obtained? A successful nursing career demands sterling qualifications—mental, moral, and physical. The duties are responsible, the discipline necessarily strict, and the hours of work onerous. Every duty that includes night work, and that involves the risk of infection cannot be otherwise accurately described than severe. Hence I regard it as a strictly business matter that women who hold high qualifications, and whose work means sacrifice, should give serious attention to their conditions of service. Any other attitude is quixotic.

### A Remarkable Phenomenon.

Trained nurses ought to have their preserves, and there ought to be a representative body always watching their class interests with jealous and vigilant eye. Doctor and lawyer have such representative bodies: so have engineers, carpenters, and bricklayers. The nurse alone, amongst large and skilled classes of workers, is an isolated unit. I regard this as a remarkable phenomenon, and at the same time as most eloquent testimony to the worthlessness of the faction-mongers who prate so loudly about their valiant doings in the past. They may belong to the old brigade, but their field of achievement is utterly barren. For the real welfare and betterment of nurses they have attempted nothing and accomplished nothing. Now, for the first time in the history of British nurses, a real union is in view, and, instead of helping, they are sparing no effort to wreck the cause. This is not sentiment, nor is it abuse: it is business—business as important to a nurse as her scale of salary. Is she to remain an isolated unit, or will she determine to form a union? The Whitley Commission recommend workers to unite, to formulate their views, and to discuss terms of remuneration and work with their employers. The average nurse does not understand these matters. She has not time to give them her serious consideration, and hence the imperative necessity for vigorous propaganda. The watchword and motto of the College of Nursing should be "Union."

The College of Nursing is not the home of a clique or of a cult. It is the potential centre of a great empire-embracing union of nurses. The conception is magnificent, but, if I may say so, a greater driving force is necessary. The individual and scattered members of the College must be inspired to immediate and vigorous pioneering. One member, one new recruit, ought to be the minimum limit of voluntary missionary effort. In my opinion this should be the effort of the rank and file rather than of the matron. The rank and file are the most directly interested. Speaking generally, the matron has achieved her ambition, and there is always the danger that her advocacy, however genuine and honest, might be misconstrued. Moreover, the hands of most matrons are more or less tied. Only within strictly defined limits would it be prudent or even possible for them to advocate the legitimate claims of their subordinates.

### An Insincere Taunt.

I know of no more hopeless and chaotic fold of nursing enterprise than Ireland. The probationer is plundered; the trained nurse is wretchedly paid; the matrons of the larger institutions are imported from Great Britain. There are no nurses' clubs, no holiday homes, no pension scheme. If the rank and file seek union at home they must be individually approved by their masters of craft, and agree to have their voting powers fixed according to a scale of eight nurses one vote. If they seek union with the College they are taunted with being anti-national and "English," an insincere taunt, seeing that the leaders of this conspiracy are themselves English or Scotch.

Every nurse should be educated to understand the dangerous forces that are working for her undoing. A nurse, as such, has no nationality, no creed, no politics. She is a woman worker whose mission of healing extends to all humanity. The glory of the Red Cross is its inherent neutrality. Wounded enemy and wounded hero engage without distinction the skill and tenderness of the Red Cross nurse. The same principle applies to the whole profession, and hence the possibility of an all-embracing union. But if this is to be achieved the need for vigorous propaganda is urgent and immediate, and it must be inspired from headquarters. Begin with union and end with union. Without this common bond all effort must necessarily be futile. When union triumphs the day of better times for working nurses will have dawned.

IERNE.

